# Spike-based Q-learning in a non-von Neumann architecture

**DOI:** 10.3389/fnins.2026.1738140

**Published:** 2026-02-03

**Authors:** Donghyuk Shin, Hyeongcheol Jo, Hyeseung Jang, Yoo Ho Jeong, YeonJoo Jeong, Joon Young Kwak, Jongkil Park, Suyoun Lee, Inho Kim, Jong-Keuk Park, Seongsik Park, Hyun Jae Jang, Hyung-Min Lee, Jaewook Kim

**Affiliations:** 1 Korea University, Seoul, Republic of Korea; 2 Korea Institute of Science and Technology (KIST), Seoul, Republic of Korea; 3 LG Electronics Inc, Seoul, Republic of Korea; 4 Ewha Womans University, Seoul, Republic of Korea

**Keywords:** non-von Neumann architecture, neuromorphic architecture, SNN, reinforcement learning, Q-learning, cart-pole

## Abstract

Non-von Neumann architectures overcome the memory-compute separation of von Neumann systems by distributing computation and memory locally, thereby reducing data-transfer bottlenecks and power consumption. These features are particularly advantageous for reinforcement learning (RL) workloads that rely on frequent value-function updates across large state-action spaces. When combined with event-driven spiking neural networks (SNNs), non-von Neumann architectures can further improve overall computational efficiency by leveraging the sparse nature of spike-based processing. In this study, we propose a hardware-feasible SNN-based non-von Neumann architecture that performs Q-learning, one of the most widely known reinforcement learning algorithms. The proposed architecture maps states and actions to individual neurons using one-hot encoding and locally stores each state–action pair's *Q*-value in the corresponding synapse. To enable each synapse to update its local *Q*-value based on the next state maximum *Q* stored in other synapses, a neuron group connected through a lateral inhibition structure is employed to produce the maximum *Q*, which is then globally transmitted to all synapses. A delay circuit is also added to align the next-state and current-state values to ensure temporally consistent updates. Each synapse locally generates a learning selection signal and combines it with the globally transmitted signals to update only the target synapse. The proposed architecture was validated through simulations on the Cart-pole benchmark, showing stable learning performance under low-bit precision and achieving comparable accuracy to software-based Q-learning with sufficient bit precision.

## 1 Introduction

Reinforcement learning (RL) provides a computational framework in which an agent learns optimal policies by interacting with the environment and receiving feedback in the form of rewards (Sutton and Barto, 2015). RL has been widely adopted in domains such as robotics, Internet of Things (IoT) systems, smart grid energy management, and communication systems, which are characterized by stringent power and latency constraints as well as the need to process large-scale streaming data efficiently (Spanò et al., 2019). To meet these requirements, researchers have focused on enhancing the computational efficiency of RL algorithms. Parallel hardware acceleration platforms, including general-purpose GPUs (Tiwari et al., 2025), field-programmable gate arrays (FPGAs; Tran et al., 2022; Salomo et al., 2025), and custom accelerators (Spanò et al., 2019), have shown substantial improvements in processing speed. Nevertheless, such approaches still exhibit much lower energy efficiency than biological neural systems, highlighting a substantial gap between artificial and biological computation (Yamazaki et al., 2022).

As an alternative to close this gap, spiking neural networks (SNNs)—a bio-plausible third-generation neural model—have attracted considerable attention (Taherkhani et al., 2020; Mehonic and Kenyon, 2022; Kiselev et al., 2025). Due to their event-driven nature, SNNs remain largely inactive in the absence of spikes, thereby enabling highly energy-efficient computation. However, executing SNN-based algorithms on conventional von Neumann architectures still suffers from computational delays and energy overhead caused by sequential memory access and control logic bottlenecks (Haşegan et al., 2022; Liu and Pan, 2023; Liu et al., 2023; Siddique et al., 2023).

Neuromorphic processors such as Intel's Loihi (Davies et al., 2018), Stanford's Neurogrid (Benjamin et al., 2014), and IBM's TrueNorth (Akopyan et al., 2015) were developed to support spike-based computation. These architectures mitigate the structural bottlenecks of von Neumann systems and demonstrate the feasibility of large-scale spike-based processing with improved energy efficiency. Recent studies have successfully implemented RL algorithms, including Deep Q-Networks (DQN) and Deep Deterministic Policy Gradient (DDPG), on the Loihi platform, thus demonstrating their potential for real-time, low-power learning (Tang et al., 2020; Akl et al., 2021; Zanatta et al., 2023).

Despite remarkable progress in neuromorphic hardware, SNN processors are not yet fully non-von Neumann architectures due to programming requirements for general-purpose functionality. For example, Loihi employs programmable virtual synaptic connections to configure neural networks with reconfigurable connectivity. Once spikes are transmitted into a core, a sequence of operations within the core—including the identification of target neurons, retrieval and update of the associated neuronal and synaptic data from memory, and storage of results—causes computational latency. Parallelization across multiple cores can alleviate memory-access delays compared with conventional von Neumann architectures; however eliminating memory-search operations altogether would enable even greater energy efficiency.

In this work, we propose a non-von Neumann architecture that performs Q-learning—a well-established reinforcement learning algorithm—based on SNNs. States and actions are one-hot encoded into input and output neurons, respectively, and the synapses between them are hardwired with a fixed topology such that each synapse locally stores and updates the *Q*(*S, A*) value through an up/down counter. This enables Q-table updates to be executed directly through spike events without requiring complex memory search or control logic, thereby reducing bottlenecks and improving energy efficiency.

A key challenge in this architecture is the distributed storage of *Q*-values across synapses, which complicates simultaneous access to both the *Q*(*S, A*) of the current state and the maximum *Q*(*S*′, *a*) of the next state. Spatially, these values are stored in different local synapses and therefore are not directly accessible, while temporally, they do not coexist immediately after a state transition. Furthermore, because the target synapse for update is not predetermined, globally transmitting the maximum *Q*(*S*′, *a*) risks unintended simultaneous updates across multiple synapses.

This challenge is addressed by proposing three architectural mechanisms. First, a population of neurons is designed to compute the maximum *Q*(*S*′, *a*) in the next state through a lateral inhibition structure, and the resulting spikes are subsequently distributed globally. Second, spikes encoding the *Q*(*S, A*) of the selected action in the current state are temporally delayed via a delay circuit to ensure their co-occurrence with the maximum *Q*(*S*′, *a*) spikes at the same time instance. Third, because spikes representing the current state and the selected action's *Q*-value are delivered simultaneously only to their corresponding synapses, their coincidence generates a selection signal that enables synapse-specific updates even in the presence of globally broadcast signals. These mechanisms enable each synapse to independently perform Q-learning updates without additional memory or address lookups.

The hardware feasibility of the proposed architecture is demonstrated through simulations in the cart-pole environment, a widely used reinforcement learning benchmark. The learning performance is further evaluated by varying the synaptic memory precision from 2 to 5 bits, allowing identification of the minimum precision required to sustain learning and the bit-width necessary to achieve performance comparable to conventional Q-learning. Such analysis provides practical insights into the trade-off between resource efficiency and learning performance in neuromorphic hardware implementations.

## 2 Background

Q-learning is a type of off-policy Temporal Difference (TD) learning, in which the value of the current state is updated using the estimated value of the next state. In off-policy learning, the behavior policy, which determines the agent's actions, is separated from the target policy that the agent aims to optimize. In Q-learning, the behavior policy is typically implemented using an epsilon-greedy policy, where an action is selected at random with probability ε, and the action that maximizes the reward is selected with probability 1−ε. The target policy, in contrast, follows a greedy policy that consistently selects the action associated with the highest *Q*-value.

The goal of Q-learning is to enable an agent to interact with its environment and learn an optimal policy that determines the best action *A* to take in each state *S*. The agent iteratively estimates the state–action value function *Q*(*S, A*), which facilitates the selection of optimal actions with respect to the current state. The Q-learning update rule is given by


$$\begin{array}{l} Q\left(S,A\right)\leftarrow Q\left(S,A\right)+\alpha \left(R+\gamma max_{a}Q\left(S^{\prime },\;a\right)-Q\left(S,A\right)\right) \end{array}$$ (1)

where α ∈ (0, 1] is the learning rate, which determines the extent to which newly obtained information overrides previously acquired estimates. *R* is the immediate reward received after taking action *A* in state *S*. The discount factor γ ∈ [0, 1] determines the relative importance of future rewards. The term $\max_{a}Q\left(S^{\prime },\;a\right)$ represents the maximum estimated value of the next state *S*′. The *Q*-value is updated after the agent performs an action *A* in the current state *S*, interacts with the environment, and subsequently observes the next state *S*′ together with the reward *R*.

## 3 Method

### 3.1 SNN architecture for Q-learning

#### 3.1.1 State-action mapping and policy implementation

**Figure 1:**
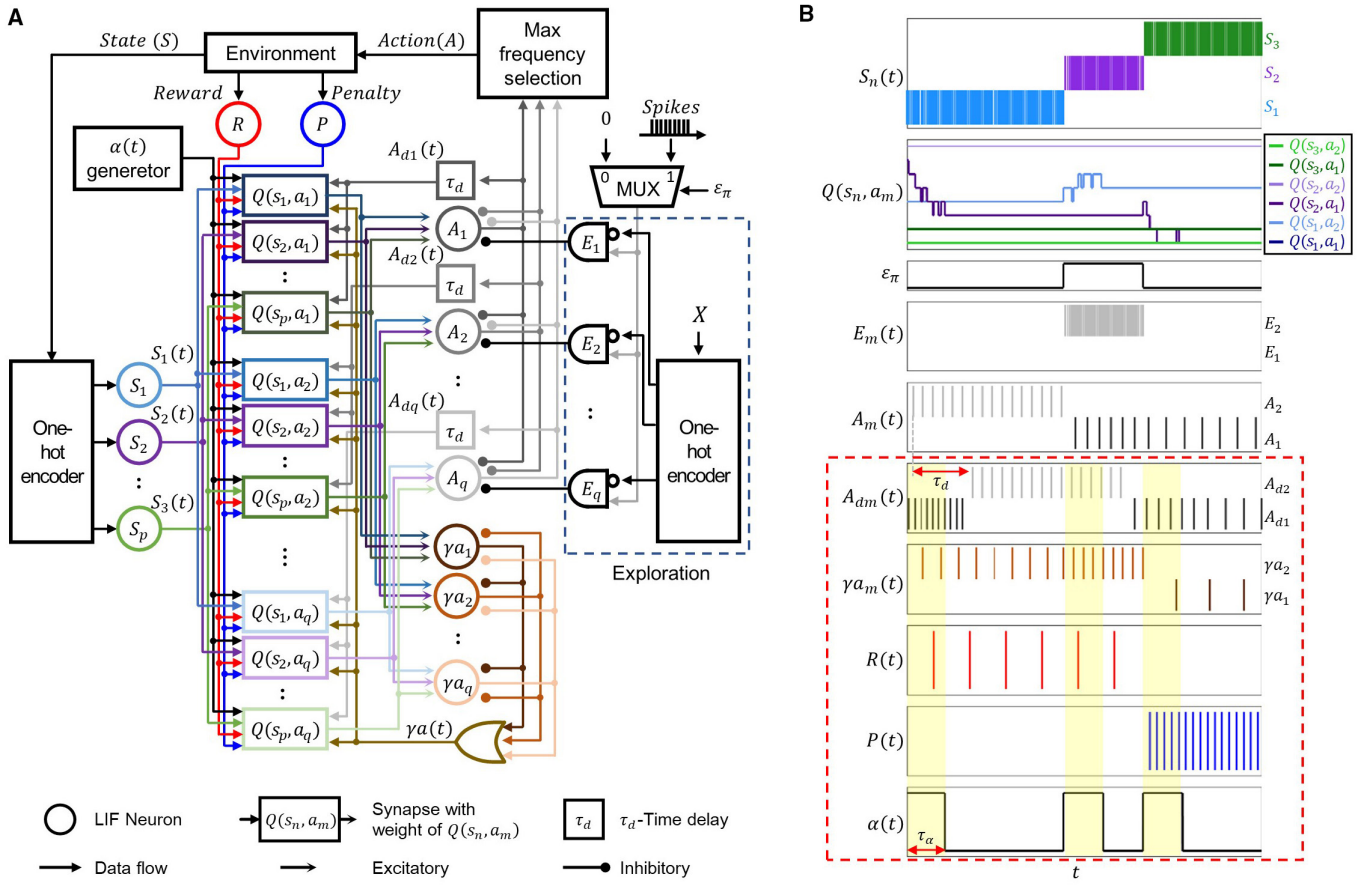
**(A)** Block diagram of the proposed non-von Neumann SNN architecture for Q-learning. **(B)** Operation waveforms of the proposed architecture for three states (*p* = 3) and two actions (*q* = 2). As the state transitions through *s*_1_, *s*_2_ and *s*_3_, the state spikes *S*_*n*_(*t*) are generated. Depending on *S*_*n*_(*t*) and the exploration signal *E*_*m*_(*t*), which is randomly activated according to ε_π_, *A*_*m*_(*t*) exhibits a firing frequency representing *Q*(*s*_*n*_, *a*_*m*_). The delayed signal *A*_*dm*_(*t*) reflects *A*_*m*_(*t*) shifted by τ_*d*_. Independent of *E*_*m*_(*t*), γ*a*_*m*_(*t*) generates spikes corresponding to the maximum *Q*(*s*_*n*_, *a*_*m*_). Based on environmental feedback, either (*t*) or (*t*) fires, and upon each state transition, α(*t*) produces a pulse of duration τ_α_.

Figure 1A shows the proposed non-von Neumann architecture implementing SNN-based Q-learning, and Figure 1B illustrates the waveforms that represent the operation of architecture. States and actions are mapped to individual leaky integrate-and-fire (LIF) neurons (Abbott, 1999), enabling a direct mapping between state-action space and neural representation. The neurons representing states and those representing actions are fully connected, and the synapses between them correspond to the Q-table, with each synaptic weight encoding *Q*(*S, A*). Each state neurons *S*_*n*_ (*n* = 1, 2, …, *p*) represents one element of the state set *S* = {*s*_1_, *s*_2_, ⋯ , *s*_*p*_}, and each action neuron *A*_*m*_ (*m* = 1, 2, …, *q*) represents one element of the action set *A* = {*a*_1_, *a*_2_, ⋯ , *a*_*q*_}.

The observed state *S* from the environment is one-hot encoded (Seger, 2018), producing a binary one-hot signal in which only the element corresponding to *S* is set to “1,” whereas all others are set to “0.” The resulting vector activates the corresponding state neuron *S*_*n*_, which in turn generates spikes transmitted to the entire population of action neurons. Under the epsilon-greedy policy, action neuron *A*_*m*_ emits spikes with a firing rate proportional to *Q*(*s*_*n*_, *a*_*m*_), determined by either exploitation or exploration.

In the proposed architecture, exploitation is implemented through a lateral inhibition structure, in which the outputs of the action neurons mutually suppress one another, allowing only the action neuron associated with the highest *Q* to become active. Exploration is implemented through the circuit shown in Figure 1A, where a discrete random variable *X* selects one element from the action set *A* = {*a*_1_, *a*_2_, ⋯ , *a*_*q*_} with uniform probability whenever the state changes. The selected value is provided as input to a one-hot encoder, which converts it into a digital parallel signal. The encoder output is then processed through an inverter, and the inverted signal is combined with the spikes generated by the MUX via an AND gate, resulting in either spikes or 0. These combined spikes suppress the action neurons before lateral inhibition takes effect, thereby allowing only the neuron corresponding to *X* to remain active and emit spikes proportional to *Q*.

The balance between exploitation and exploration is determined by the discrete random variable ε_π_, which takes the value 0 with probability 1−ε and 1 with probability ε. When ε_π_ = 0, the MUX output is 0, and the architecture operates in exploitation mode without suppression of the action neurons by the AND gates. Conversely, when ε_π_ = 1, the MUX output generates spikes that pass through the AND gates and suppresses all but one action neuron, thereby enabling exploration. The outputs of the action neurons are subsequently transmitted to a selection module that identifies the action neuron with the highest firing frequency and delivers the corresponding action *A* to the environment.

Figure 1B illustrates the spiking activity of the state neurons in response to state transitions and the spiking of the action neurons as determined by the value of ε_π_ for *p* = 3 and *q* = 2. The state changes asynchronously in the order of *s*_1_, *s*_2_, *s*_3_, causing the corresponding *S*_1_, *S*_2_, and *S*_3_ neurons to fire sequentially. After each state transition, *Q*(*s*_*n*_, *a*_*m*_) is immediately updated and details on this *Q* update process are provided in Section 3.2. For *s*_1_ and *s*_3_, where exploitation is applied, the *A*_2_ and *A*_1_ neurons fire according to the highest *Q*, *Q*(*s*_1_, *a*_2_) and *Q*(*s*_3_, *a*_1_), respectively. In contrast, for *s*_2_, where exploration is applied, the *A*_1_ neuron fires despite *Q*(*s*_2_, *a*_2_) being higher than *Q*(*s*_2_, *a*_1_), due to the suppression of the *A*_2_ neuron by the *E*_2_ spikes.

#### 3.1.2 Spike encoding for Q-learning updates

To adapt Q-learning updates for SNN operation, the elements of Equation 1, which include the reward *R*, $\gamma max_{a}Q\left(S^{\prime },\;a\right)$, and *Q*(*S, A*), are encoded as spike signals whose firing frequencies are proportional to their respective values and delivered to the target synapses. The learning rate α is represented as a pulse whose width is proportional to the corresponding value and is transmitted to all synapses. The encoding and delivery of these transformed signals are illustrated in the red dashed box in Figure 1B.

Spike signals with firing frequencies proportional to *Q*(*S, A*) and $\max_{a}Q\left(S^{\prime },\;a\right)$ can be generated by introducing LIF neurons driven by these terms. In the proposed architecture, however, the firing frequency of each action neuron is inherently proportional to *Q*(*S, A*). Therefore, spike signals representing *Q*(*S, A*) can be obtained directly from the existing action neurons without the need for additional circuitry (Figure 1A).

When exploration is applied under the epsilon-greedy policy, spike signals with firing frequencies proportional to $\max_{a}Q\left(S^{\prime },\;a\right)$ is not obtainable from the action neurons. To address this limitation, the proposed architecture incorporates additional γ*a*_*m*_ neurons (Figure 1A). These neurons share the same synaptic connections as the action neurons but implement only the lateral inhibition structure associated with exploitation. The scaling factor γ is determined by adjusting the thresholds of the γ*a*_*m*_ neurons, and their firing frequencies vary in proportion to γ.

For the Q-learning update, both the *Q*-value of the current state and that of the next state are required to be simultaneously available. However, because only the current *Q*-value is stored in synapses, the *Q*-value of the next state becomes available only after the state transition. To resolve this issue, the proposed architecture incorporates a delay mechanism that enables the coexistence of the current and next *Q*-values within the next state by delaying the *A*_*m*_ spikes corresponding to *Q*(*S, A*). Specifically, the spike signals *A*_*m*_(*t*), which represent to *Q*(*S, A*), are delayed by a fixed time interval τ_*d*_ to generate *A*_*dm*_(*t*). This delayed signal ensures that, during the next state, spikes representing *Q*(*S, A*) remain available for a duration of τ_*d*_.

The outputs of each *A*_*m*_ neuron are delayed individually, such that spikes corresponding to *Q*(*S, A*) can be delivered to the synapses of the same *A*_*m*_ neuron during the next state, thereby enabling the Q-learning update. The outputs of the γ*a* neurons are combined using an OR gate to form a single γ(*t*) signal, which is then delivered to all synapses.

In Figure 1B, spikes corresponding to *Q*(*S, A*) and spikes corresponding to $\gamma max_{a}Q\left(S^{\prime },\;a\right)$ coexist for a duration of τ_*d*_ immediately following a state transition. For example, after the transition from *s*_1_ to *s*_2_, *A*_*d*2_ spikes appear with a firing frequency proportional to *Q*(*s*_1_, *a*_2_), while, within the same interval, γ*a*_2_ spikes emerge with a frequency proportional to *Q*(*s*_2_, *a*_2_ ).

The reward signal *R*, positive for rewards and negative for penalties, is converted into spikes without sign information by introducing two additional neurons (Figure 1A): an *R* neuron for rewards and a *P* neuron for penalties. For each state, when a reward occurs, only the *P* neuron emits spikes with a frequency proportional to the reward magnitude, whereas when a penalty occurs, only the *P* neuron emits spikes with a frequency proportional to the penalty magnitude. The spikes from theses neurons are delivered to all synapses to drive the Q-learning update.

The spike signals corresponding to the terms in Equation 1 coexist only during a limited interval τ_*d*_ after a state transition, which defines the effective update window in the next state. In the proposed architecture, the learning rate α is implemented by an α generator that produces a pulse of width τ_α_, proportional to α and bounded by τ_*d*_. This pulse is triggered at each state transition and only spikes occurring within the τ_α_ window contribute to the Q-learning updates. A smaller τ_α_ results in fewer spikes being involved in the computation. As illustrated in Figure 1B, when τ_α_ < τ_*d*_, only *R, P*, *A*_*dm*_ and γ*a*_*m*_ spikes within the τ_α_ window are utilized for learning.

#### 3.1.3 Spike-based synaptic update circuit for Q-learning

As shown in Figure 1A, *A*_*dm*_(*t*), *R*(*t*), *P*(*t*), and γ*a*(*t*) are delivered globally to all synapses. *S*_*n*_(*t*) is transmitted only to the synapses connected to the specific state neuron *S*_*n*_, while *A*_*dm*_(*t*) is transmitted exclusively to the synapses connected to the selected action neuron *A*_*m*_. Consequently, both signals are simultaneously present only at synapses where the current state and the currently selected action are jointly represented. The proposed architecture exploits this structural feature to generate a selection signal based on these two inputs, which in turn determines the Q-learning update.

**Figure 2:**
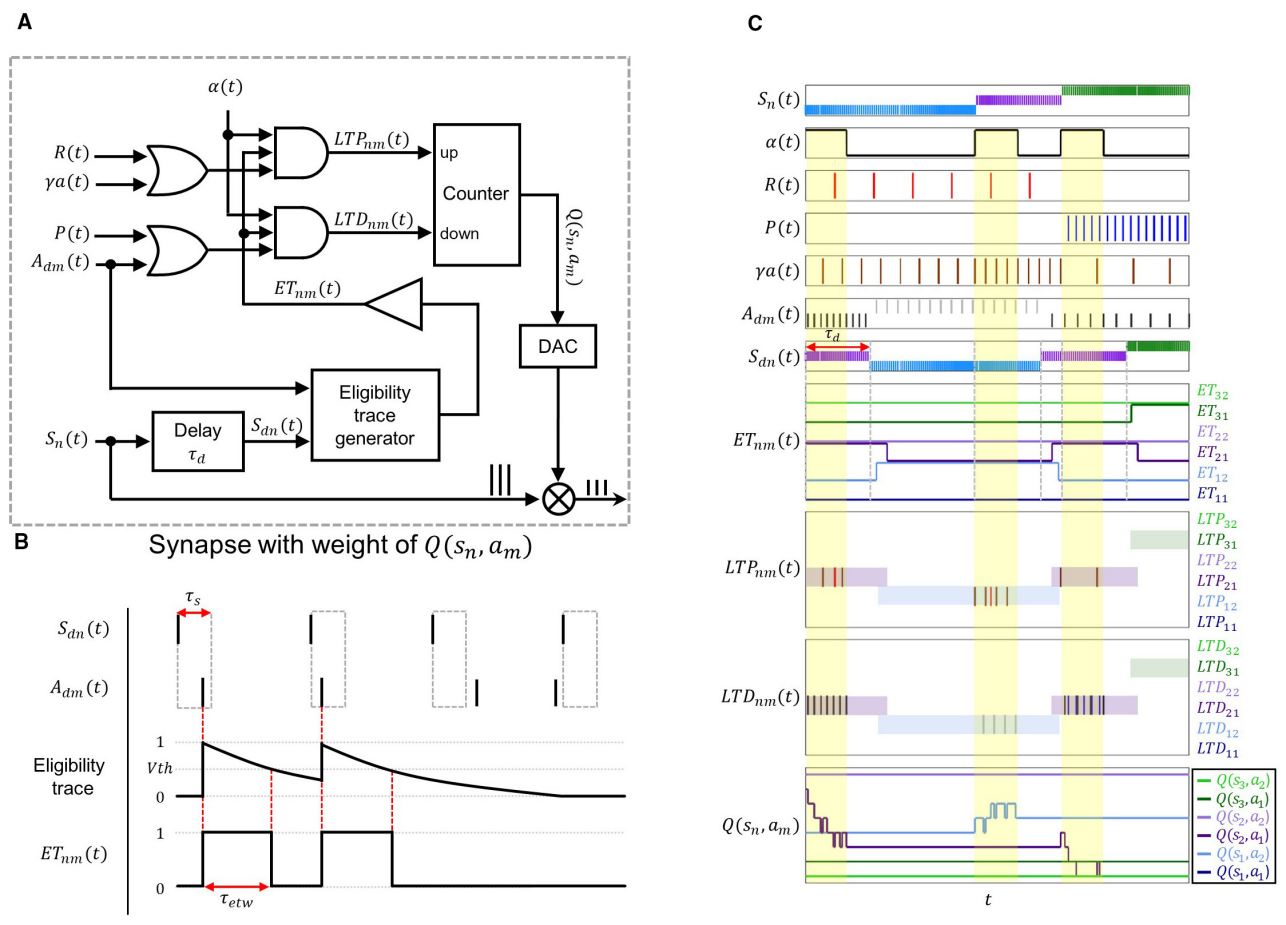
**(A)** Block diagram of a synaptic circuit performing local updates of the weight corresponding to *Q*(*s*_*n*_, *a*_*m*_), where the delayed state and delayed action spikes generate an eligibility trace that is combined with update-related inputs to produce *LTP*_*nm*_/*LTD*_*nm*_ spikes driving the counter-based *Q*-value update. **(B)** Operation waveforms of the eligibility trace generator and the waveform conversion of the trace using a buffer. The eligibility trace is generated when an *A*_*dm*_ spike occurs within τ_*s*_ after an *S*_*dn*_ spike, and this trace is converted into the *ET*_*nm*_ pulse of duration τ_*etw*_ through a buffer with a threshold *V*_*th*_. **(C)** Operation waveforms illustrating the *Q*(*s*_*n*_, *a*_*m*_) update process based on signals transmitted to and generated within the synaptic block. *ET*_*nm*_(*t*) pulses are generated when the delayed state signal *S*_*dn*_(*t*), obtained by shifting *S*_*n*_(*t*) by τ_*d*_, coincides with *A*_*dm*_(*t*). When the yellow-shaded α(*t*) pulse overlaps with *ET*_*nm*_(*t*) pulses, *LTP*_*nm*_(*t*) spikes are induced by (*t*) and γ*a*(*t*), whereas *LTD*_*nm*_(*t*) spikes are induced by *P*(*t*) and *A*_*dm*_(*t*). Each *LTP*_*nm*_(*t*) and *LTD*_*nm*_(*t*) spike updates *Q*(*s*_*n*_, *a*_*m*_) by a single step.

Figure 2A shows the block diagram of an individual synapse in the proposed architecture, which performs the computations required for Q-learning update and stores the *Q*-values. As Q-learning updates occur during the τ_*d*_ period of the next state, the *S*_*n*_ spikes are delayed by τ_*d*_ to remain valid within this interval. After the occurrence of *S*_*dn*_ spikes, the subsequent arrival of *A*_*dm*_ spikes generate the eligibility trace.

The eligibility trace generator can be realized using a capacitor–MOSFET structure, in which capacitors integrate incoming spikes and discharge gradually through leakage, while a MOSFET gates the signal according to the resulting voltage. This circuit configuration produces a decaying trace that represents synaptic eligibility (Wijekoon and Dudek, 2011). The resulting trace is converted by a buffer into an *ET*_*nm*_ pulse of duration τ_*etw*_ (Figure 2B), defining the time window in which learning is valid. As illustrated in Figure 2C, *ET*_*nm*_(*t*) is generated at the synapse corresponding to *Q*(*S, A*) during the τ_*d*_ period of the next state. After the transition from *s*_1_ to *s*_2_, the *S*_*d*1_ spikes from the previous state *s*_1_ and the *A*_*d*2_ spikes generate an *ET*_12_ pulse that remains HIGH for the duration of τ_*d*_, enabling only *Q*(*s*_1_, *a*_2_) to be updated in state *s*_2_.

The up/down counter is employed to store *Q*(*s*_*n*_, *a*_*m*_) values and to update them using spike-based signals (Figure 2A). The input spikes are generated by classifying the signals in Equation 1 into those that increase *Q*(*s*_*n*_, *a*_*m*_) and those that decrease it, and combining each group with logic gates. Specifically, (*t*) and γ(*t*) are grouped for potentiation, and *P*(*t*) and *A*_*dm*_(*t*) are grouped for depression, with each pair combined through OR gates. The outputs of the OR gates are subsequently gated by (*t*) and *ET*_*nm*_(*t*) using AND operations, producing *LTP*_*nm*_(*t*) and *LTD*_*nm*_(*t*) signals.

The up/down counter receives *LTP*_*nm*_ spikes at its up input, increasing *Q*(*s*_*n*_, *a*_*m*_) by one count per spike, and *LTD*_*nm*_ spikes at its down input, decreasing *Q*(*s*_*n*_, *a*_*m*_) by one count per spike. Figure 2C shows the synaptic updates of *Q*(*s*_*n*_, *a*_*m*_) driven by spikes in the proposed architecture. Within the time window where the α pulse and the *ET*_*nm*_ pulse coexist, *LTP*_*nm*_ spikes are generated from the combination of *R* spikes and γ*a* spikes, while *LTD*_*nm*_ spikes arise from the combination of *P* spikes and *A*_*dm*_ spikes. Each occurrence of an *LTP*_*nm*_ spike results in a real-time increase in *Q*(*s*_*n*_, *a*_*m*_), whereas each *LTD*_*nm*_ spike results in a real-time decrease.

### 3.2 Cart-pole task environment

**Figure 3:**
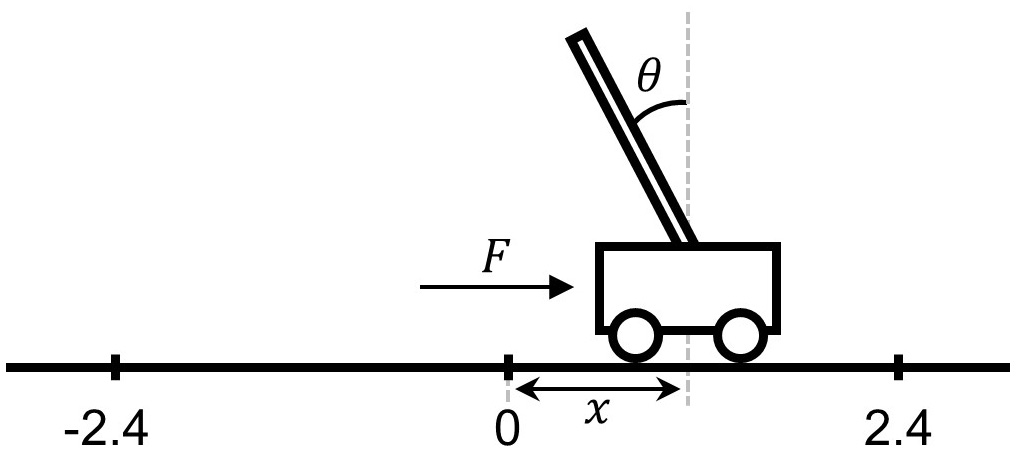
Cart-pole game environment.

The cart-pole task, illustrated in Figure 3, is a standard benchmark in reinforcement learning where a force is applied to a cart along the x-axis on a flat surface with the goal of maintaining the pole balanced on the cart (Geva and Sitte, 1993). In this study, simulations were conducted using the cart-pole environment provided in the Reinforcement Learning Toolbox of MATLAB. Each episode was initialized with the cart positioned at the origin and the pole in an upright orientation. At every 20 ms time step, a force of either +10 N or 10 N was applied to the cart. An episode terminates in failure if the cart position exceeds ±2.4 units from the origin or if the pole angle exceeds ±12 °. Conversely, an episode is considered successful if the pole remained balanced within these bounds for 4 s.

The state variables of the cart-pole environment are the cart position *x*, cart velocity ẋ, pole angle θ, and pole angular velocity $\dot{\theta }$. These variables were quantized as follows:


$$\begin{array}{r} x:\left(-2.4,2.4\right);ẋ:\left(-\infty ,\infty \right);\\ \theta :\left(-12,\;-0.1\right],\;\left(-0.1,-0.01\right],\;\left(-0.01,\;0\right],\;\left(0,\;0.01\right],\\ \left(0.01,\;0.1\right],\;\left(0.1,\;12\right);\\ \dot{\theta }:\left(-\infty ,\;-0.87\right],\;\left(-0.87,\;0.87\right],\;\left(0.87,\infty \right). \end{array}$$

The state set *S* consists of 19 elements, comprising 18 four-dimensional tuples from the combinations of the state variables and one failure state of the cart-pole task. Within the action set *A* = {−10 N, 10 N}, the proposed architecture contained 38 synapses encoding the corresponding *Q*(*s*_*n*_, *a*_*m*_) values. For non-failure states *s*_1_- *s*_18_, a reward of +1 is assigned, whereas for the failure state *s*_19_, a penalty of −8 is applied. The parameter ε in the epsilon-greedy policy, which determines the probability of exploitation and exploration, was initialized at 1 and decays by a factor of 0.7 across episodes.

## 4 Experiments & results

To evaluate the operation of the proposed non-von Neumann architecture in a hardware-oriented context, a high-level simulation model was implemented in MATLAB and interfaced with the cart-pole environment. All simulations were performed on a workstation with an Intel(R) Core™ i7-8700 CPU @ 3.20 GHz and 16 GB of RAM.

In the simulations, the model parameters were set as follows: the learning rate α = 1, the discount factor γ = 0.99, a counter bit-width of 3 bits, a reward of +1, and a penalty of −8. The firing frequency of the state neurons was fixed at 10 kHz, whereas the action neurons fired at frequencies ranging from 201 to 1,610 Hz depending on the *Q*-values stored in their corresponding synapses. The eligibility trace window τ_e*tw*_ was set to 14 ms to ensure that, at the lowest action neuron frequency of 201 Hz, the trace generated by an *A*_*dm*_ spike persisted in the buffer until the next spike arrived. A detailed summary of the simulation parameters is provided in Table 1.

**Table 1:** Model parameters used in the simulation of the proposed architecture.

**Parameter**	**Value**
Bit-width	3-bit
*S*_*n*_ freq (Hz)	10k
τ_*d*_ (ms)	5
τ_α_ (ms)	5
Reward freq (Hz)	205
Penalty freq (Hz)	1,700
τ_*etw*_ (ms)	14

**Figure 4:**
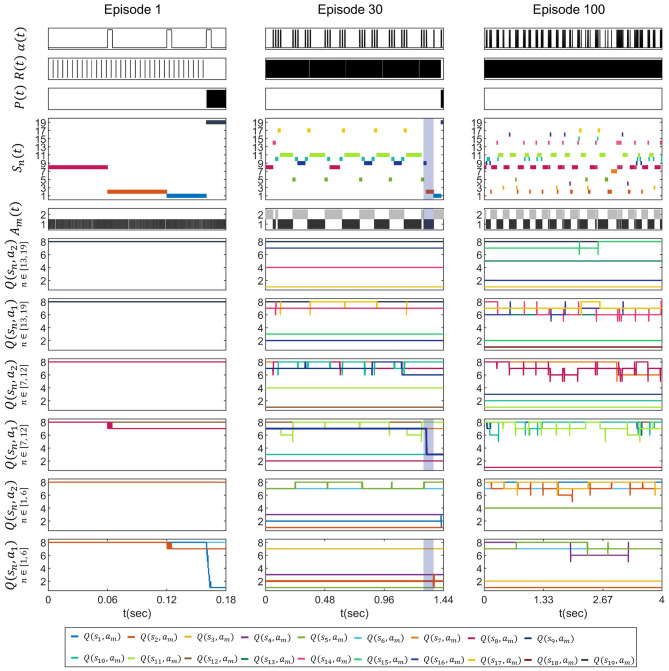
Simulation results of the cart-pole task for episodes 1, 30, and 100, showing failures at 0.18 s and 1.94 s in episode 1 and 30, and successful balance at 4 s in episode 100. Each panel shows the learning rate pulse (*t*), the reward spikes (*t*), the penalty spikes *P*(*t*), the state spikes *S*_*n*_(*t*) for n = 1, 2, …, 19, the action spikes *A*_*m*_(*t*) for *m* = 1, 2, and the 3-bit quantized *Q*-values *Q*(*s*_*n*_, *a*_*m*_), represented using integer levels from 1 to 8. The *Q*-value trajectories are shown in separate *Q*(*s*_*n*_, *a*_1_) and *Q*(*s*_*n*_, *a*_2_) panels, with each panel corresponding to a different subset of states (*s*_1_*s*_6_, *s*_7_*s*_12_, and *s*_13_*s*_19_).

Based on these parameters, we evaluated the proposed architecture in the cart-pole environment across 100 episodes. Figure 4 shows the simulated waveforms of Episodes 1, 30, and 100. The signals *R*(*t*), *P*(*t*), γ*a*(*t*), and *A*_*dm*_(*t*) denote spike trains over time, whereas *Q*(*s*_*n*_, *a*_1_) and *Q*(*s*_*n*_, *a*_2_) represent the corresponding *Q*-values, updated in time and quantized to 3 bits. The colors of the *Q*(*s*_*n*_, *a*_1_) and *Q*(*s*_*n*_, *a*_2_) traces are matched to those of the corresponding *S*_*n*_ spikes to indicate correspondence.

In episode 1, the *Q*(*s*_*n*_, *a*_*m*_) values were initialized to their maximum. Since all *Q*(*s*_*n*_, *a*_*m*_) values were identical at the start, most of them changed only slightly during learning. However, once the state transitioned to the failure state *s*_19_, *Q*(*s*_18_, *a*_1_) decreased sharply in response to the *P* spikes, leading to the termination of the episode.

In episode 30, the initial *Q*-values reflected the learning accumulated from previous episodes. Within the green-shaded interval between 0.96 s and 1.44 s, the state transitioned from *s*_9_ to *s*_2_, with the action *A*_1_ selected in both states. At *s*_9_, the *Q*(*s*_9_, *a*_1_), shown by the thick blue trace, corresponds to *Q*(*S, A*), whereas at *s*_2_, the *Q*(*s*_2_, *a*_1_), shown by the thick orange line, corresponds to $\max_{a}Q\left(S^{\prime },\;a\right)$.The Q-learning update defined in Equation 1 was executed, causing *Q*(*s*_9_, *a*_1_) to decrease by four steps. Similar to episode 1, episode 30 also terminated when the state reached the failure state *s*_19_ at 1.44 s.

In episode 100, the simulation terminated successfully after maintaining balance for the full 4 s without entering the failure state *s*_19_. The *Q*(*s*_*n*_, *a*_*m*_) had stabilized and, apart from minor deviations of approximately one step following updates, remain largely unchanged from their prior values.

The performance of the proposed architecture was evaluated by averaging scores every 20 episodes across 10 independent simulation runs. The score increased by 1 for every 20 ms in which the pole remained balanced, reaching a maximum of 200 when balance was maintained for 4 s.

**Figure 5:**
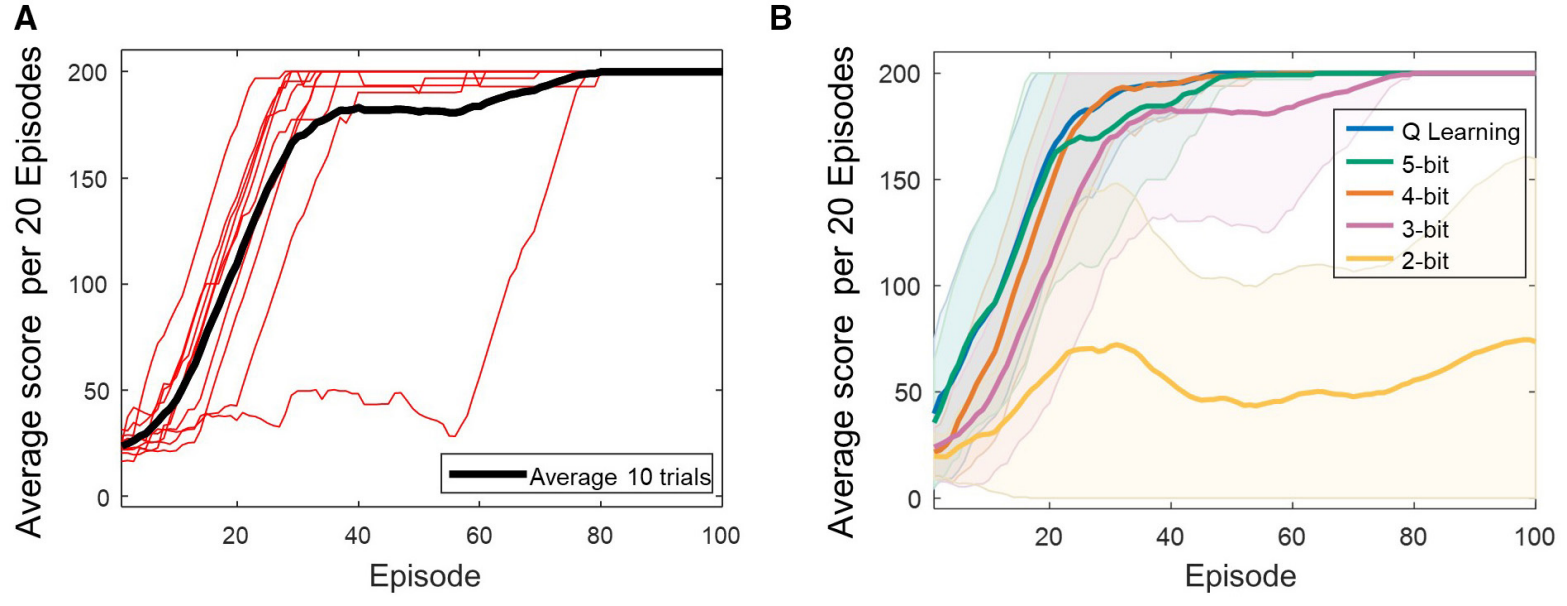
**(A)** Learning curves obtained using a 3-bit counter in the proposed architecture. Red lines indicate the average score per 20 episodes for each of the 10 trials, and the black line shows the overall mean. **(B)** Comparison of average score per 20 episodes across different counter bit-widths: 5-bit (green), 4-bit (orange), 3-bit (pink), and 2-bit (yellow), and standard Q-learning (blue). The solid lines show the average score per 20 episodes over 10 trials, and the shaded area represent the standard deviation.

The red traces in Figure 5A show the average score per 20 episodes for each of the 10 simulations conducted with a 3-bit counter, while the black trace shows the mean of these averages across simulations. Although individual runs vary due to exploration governed by the epsilon-greedy policy, the results indicate that the average score reaches 200 within 100 episodes.

Figure 5B compares the average score per 20 episodes across 10 simulations with α = 1 and γ = 0.99, under the counter bit-widths of 2, 3, 4, and 5, as well as conventional Q-learning without bit limitations. The experimental parameters for each counter bit configuration are summarized in Table 2. In this experiment, the parameters for each bit-width configuration were selected to ensure stable operation of the architecture. The reward was fixed at the minimum unit of +1, while the penalty was set to the maximum negative value representable by each bit-width. Furthermore, the frequencies of the reward and penalty signals were adjusted so that the number of spikes associated with each value was appropriately reflected within the maximum valid time window τ_*d*_.

**Table 2:** Parameters for different counter bit widths in the proposed architecture.

**Parameter**	**Value**			
Bit-width	2-bit	3-bit	4-bit	5-bit
*S*_*n*_ freq (Hz)	10k	10k	20k	40k
τ_*d*_ (ms)	2	5	8	10
τ_α_ (ms)	2	5	8	10
Reward freq (Hz)	505	205	127	105
Penalty freq (Hz)	2,200	1,700	2,050	3,250
τ_*etw*_ (ms)	17	14	11	9

With the 2-bit counter (yellow trace), the cart-pole task failed as the average score does not reach 200. The 3-bit counter (pink trace) achieved success approximately 40 episodes later than conventional Q-learning (blue trace), whereas the 4-bit (orange trace) and 5-bit (green trace) counters reached an average score of 200 within about 50 episodes, comparable to Q-learning. These results demonstrate that the proposed architecture can successfully solve the cart-pole task with a 3-bit counter, while performance comparable to Q-learning is obtained with a 4-bit counter.

The performance graph in Figure 5B, generated using the parameters listed in Table 2, was analyzed using a one-way analysis of variance (ANOVA), and the results are summarized in Table 3. A statistically significant effect of quantization level on performance was observed [_(4, 45)_ = 60.0544, *p*-value < 0.0001], encompassing unquantized Q-learning and 2–5-bit representations. Subsequently, Tukey's honestly significant difference (HSD) *post-hoc* tests were performed to compare Q-learning with each bit-width and the results are presented in Table 4. *Post-hoc* analyses revealed no significant differences between Q-learning and the 5-bit, 4-bit, or 3-bit models (all *p*-values ≥ 0.987). In contrast, the 2-bit condition showed significantly lower performance compared with Q-learning (*p*-value < 0.0001).

**Table 3:** One-way ANOVA across quantization levels.

**Source**	**SS**	***df***	**MS**	***F*-value**	***p*-value**
Quantization level	160,810	4	40,204	60.0544	< 0.0001
Error	30,125	45	669.45		
Total	190,940	49	8		

**Table 4:** Tukey's HSD *post-hoc* comparisons between Q-learning and models with different bit-widths.

**Comparison**	**Mean diff**	**95%**	***p*-value**
Q-learning−5-bit	−0.0360	[−32.9148, 32.8428]	1.0000
Q-learning−4-bit	0.2700	[−32.6088, 33.1488]	1.0000
Q-learning−3-bit	5.7375	[−27.1413, 38.6163]	0.9874
Q-learning−2-bit	143.1677	[110.2889, 176.0465]	< 0.0001

## 5 Discussion

In this study, we proposed a non-von Neumann SNN architecture specialized for the Q-learning algorithm. The proposed system employs a hard-wired connectivity with a fixed network topology, in which each synapse stores a single *Q*-value, thereby reducing memory-access overhead through localized storage. This architectural approach contrasts with general-purpose neuromorphic processors such as Intel's Loihi, which adopt reconfigurable neural connectivity to support various network topologies but typically involve centralized or shared memory access, potentially leading to memory-access bottlenecks within the core. In this context, this work emphasizes algorithm-hardware co-optimization rather than hardware reconfigurability, suggesting a promising direction for improving computational efficiency. This approach aligns with prior studies emphasizing the need for co-design across multiple levels of neuromorphic systems—including hardware, circuits, algorithms, and applications (Schuman et al., 2022)—and suggests the potential of algorithm-centered hardware specialization as a direction for future neuromorphic hardware development.

These architectural differences are reflected in the energy efficiency and area characteristics. In term of energy efficiency, the synaptic weights in Loihi are stored in SRAM, and each spike is processed through AER address decoding, synapse selection, memory access, and a read–modify–write update, with the spike delivered as a packet across the on-chip network. While this packet-based event-driven approach is highly efficient for sparse activity, the dynamic power consumed per spike can increase as spike events are transmitted in packet form. In contrast, in the proposed architecture, each *Q*-value is stored in a local counter and spikes are routed directly through fixed wiring, thereby avoiding packet conversion and address decoding and reducing the amount of data movement and the activation of update-related circuitry.

In terms of area, Loihi is designed such that the neurons and synapses within each core share a common computation and learning engine, whereas in the proposed architecture, dedicated processing units and local learning circuits are assigned to each neuron and synapse block. As a result, Loihi can achieve a relatively higher neuron and synapse density per unit area. However, when the full system architecture is considered, Loihi includes additional blocks such as the network on chip (NoC), AER interface logic for packet processing, and routers, which contribute non-negligibly to the overall chip area. By comparison, although separate blocks for the NoC and packet-based routing are not required in the proposed architecture, additional area overhead arises from the increased wiring needed for the fixed connectivity between neuron and synapse blocks. The practical impact of these factors in implementation will require further examination and careful evaluation.

Another notable aspect of the proposed architecture is its alignment with biological learning processes observed in the brain. In the proposed system, distributed computation occurs locally at each synapse, global reward signals are broadcast throughout the network, and synapse-specific learning is achieved through local signal generation—analogous to the interplay between global modulatory signals and local synaptic events in the brain. In the brain, slow global signals such as hormones or neuromodulators regulate long-term learning, while local spike interactions at specific synapses drive plasticity (Brzosko et al., 2019). Similarly, the proposed architecture globally propagates both the reward and $\max_{a}Q\left(S^{\prime },\;a\right)$, and generates local selection signals through the coincidence of pre- and post-synaptic spikes corresponding to state–action pairs. Moreover, the delay mechanism introduced to address temporal mismatches aligns with biological timing characteristics. Neural systems exhibit axonal conduction delays (Madadi Asl et al., 2017), synaptic transmission delays, and recurrent-circuit delays, all of which play crucial roles in learning mechanisms such as spike-timing-dependent plasticity (STDP). These similarities suggest that the proposed non-von Neumann architecture captures key functional aspects of biological learning mechanisms.

From a hardware perspective, this study demonstrated that a minimal 3-bit precision up/down counter used as a synaptic memory was sufficient to complete the cart-pole simulation within 100 episodes, confirming the feasibility of low-precision memory in practical learning. As the architecture scales, the number of synapses (*p*×*q*) grows much faster than the number of neurons (*p*+2*q*), making synaptic memory bit width and area efficiency critical constraints in hardware design. Therefore, the finding that stable learning can be achieved with as few as 3 bits supports the practical feasibility of implementing the proposed architecture on neuromorphic hardware.

Beyond precision considerations, it is also important to assess whether the proposed architecture remains robust when scaled to larger network sizes. In conventional von Neumann systems, *Q*-values are stored in centralized memory, requiring frequent memory accesses and substantial data movement during learning. Consequently, memory bottlenecks have been a major limitation when such systems are scaled. In contrast, in the proposed architecture, *Q*-values are stored in local counters within each synapse block, and learning is carried out in parallel across synapses, so that memory-related bottlenecks do not arise structurally during scaling.

A remaining concern in large-scale expansion is whether propagation delays along long signal routes could introduce timing mismatches in learning. In the proposed architecture, learning is based on counting spikes within an α pulse of duration τ_α_, with a maximum timing tolerance defined by τ_*d*_. Global update-related signals—such as *S*_*n*_(*t*), *A*_*dm*_(*t*), γ*a*(*t*), *R*(*t*), and *P*(*t*)—are routed across synapse blocks through wires of varying lengths. Differences in wire lengths can introduce arrival-time variations, which may affect the number of spikes captured within the α pulse and lead to non-uniform Q-updates across the network. In the presented 3-bit implementation, update-related signals operate at a maximum frequency of 10 kHz, such that a 1% timing variation corresponds to approximately 1 μs. In 16–22 nm technology nodes, the reported propagation delay is about 2 ns per millimeter [International Technology Roadmap for Semiconductors (ITRS), 2007]. At this rate, a delay of 1 μs would accumulate only over wire lengths exceeding approximately 555 mm, which is far beyond the dimensions of a typical single chip. Even in multi-chip board-level configurations, substantial margin therefore remains before routing-induced delays would meaningfully affect learning behavior.

In addition to these hardware-level considerations, large-scale Q-learning presents challenges, particularly in terms of slower convergence and reduced generalization when the state–action space becomes very large. As the number of states increases, experience becomes sparsely distributed across the space, reducing opportunities for repeated correction of specific situations. This sparsity slows learning and can lead to generalization errors in which the agent assigns inaccurate *Q*-values to insufficiently explored states. As the dimensionality of the environment grows, these challenges become more severe, often requiring substantially more interactions to achieve stable learning outcomes. In future work, large-scale simulations may be used to evaluate the impact of update sparsity on performance, and concepts inspired by similarity-based update approaches (Rosenfeld et al., 2017) may be incorporated to ensure that related state–action pairs reflect the most recent environmental information even under sparse updates. Additionally, the proposed architecture incorporating these approaches may also be implemented on neuromorphic hardware.

## Data Availability

The original contributions presented in the study are included in the article/supplementary material, further inquiries can be directed to the corresponding author.
